# CT‐guided microwave ablation in patients with lung metastases from breast cancer

**DOI:** 10.1111/1759-7714.14212

**Published:** 2021-11-02

**Authors:** Min Meng, Xiaoying Han, Wenhong Li, Guanghui Huang, Yang Ni, Jiao Wang, Tiehong Zhang, Jianjian Dai, Zhigeng Zou, Xia Yang, Xin Ye

**Affiliations:** ^1^ Department of Oncology Shandong Provincial Hospital Affiliated to Shandong First Medical University Jinan China; ^2^ Department of Oncology The First Affiliated Hospital of Shandong First Medical University Jinan China

**Keywords:** breast cancer, CT‐guided, lung metastasis, microwave ablation

## Abstract

**Background:**

Computed tomography (CT)‐guided percutaneous microwave ablation (MWA) is a very common ablation method that shows a good local tumor control rate in primary and secondary lung tumors. At present, few reports have explored the safety and efficacy of MWA for lung metastases from breast cancer.

**Methods:**

From January 2012 to January 2018, 32 breast cancer patients with 46 pulmonary metastases received CT‐guided percutaneous MWA. The study was approved by the local institutional review board. The clinical efficacy and complications of MWA were investigated.

**Results:**

The median follow‐up time was 32 months and the main effective rate was 97.8% (45/46). Five of 46 lesions had local progression (10.9%), with a median progression time of 10 months. The 1‐, 3‐, and 5‐year overall survival (OS) rates were 96.9%, 53.3%, and 17.8%, respectively. The median OS time was 36 months. Among 46 MWA treatments, 11 (23.9%) had massive pneumothorax, two (4.3%) had massive pleural effusion, and two (4.3%) had a pulmonary infection.

**Conclusion:**

CT‐guided percutaneous MWA may be safe and effective for treating lung metastases from breast cancer.

## Graphical abstract

Most patients with lung metastases from breast cancer, especially those with multiple metastases, are ineligible for operation. Thirty‐two patients who underwent microwave ablation (MWA) for lung metastases from breast cancer over a 6‐year period were examined. Computed tomography‐guided percutaneous MWA may be safe and effective for treating lung metastases from breast cancer.

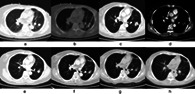

## INTRODUCTION

Breast cancer is one of the most common tumors in women and the second most common in estimated deaths. Epidemiological studies showed that 20–50% of patients with early breast cancer had distant metastasis within 5 years.^1^ The lung is the common site of distant metastasis. The 5‐year survival rate of metastatic breast cancer patients is about 20%.^1^, ^2^ Among these metastatic patients, lung metastasis is associated with the worst prognosis. Metastatic breast cancer to the lungs is considered a systemic disease. Even with systemic chemotherapy, hormonal therapy, or both, the median survival time reported is only 22.5 months.^3^ Although there are many methods to treat lung metastasis, such as chemotherapy, radiotherapy, and targeted therapy, the survival rate of breast cancer patients with lung metastasis is still very low.^4^, ^5^

For solitary lung metastases, surgical resection can be used. However, the role of surgical resection of lung metastases is still not generally accepted^6^, ^7^; moreover, for patients with multiple lung metastases, comprehensive treatment should be the priority. In recent years, some minimally invasive surgeries for tumors have developed rapidly, such as microwave ablation (MWA), cryotherapy, laser therapy, and radiofrequency ablation (RFA).^8^, ^9^ Ablation of lung metastases has important potential advantages, including selective lung injury and repeatability of treatment.^10^ The ultimate goal of ablation is to prolong the survival time and make the tumor fully controlled.

MWA is a very common ablation method that shows a good local tumor control rate in primary and secondary lung tumors. It has the advantages of a shorter operation time, a larger volume of necrosis in the ablation area, and less “heat sink” effect, making more patients with malignant tumors accept MWA as an alternative. MWA usually uses 915 or 2450 MHz. In the microwave electromagnetic field, water molecules, protein molecules, and other polar molecules in tumor tissue will vibrate at high speed, resulting in a molecular collision and mutual friction. This will produce a temperature of 60–150°C.^11^ MWA for inoperable pulmonary malignancies is one of the most popular minimally invasive techniques in recent years.^12^

At present, there are few studies on MWA for lung metastases from breast cancer. As outlined above, this study aimed to investigate the local control and long‐term efficacy of MWA for lung metastases from breast cancer.

## MATERIALS AND METHODS

### Patients

This study was approved by the Institutional Ethics Committee of Shandong Provincial Hospital Affiliated of Shandong First Medical University. Written informed consent from each patient was obtained before the operation.

To evaluate the efficacy of MWA for the local control and long‐term efficacy of lung metastases from breast cancer, inclusion and exclusion criteria were established. (1) The number of unilateral lung lesions was ≤3 (bilateral lung lesions ≤5), the maximum diameter of multiple metastases was ≤3 cm, and the maximum diameter of single unilateral metastasis was ≤5 cm. (2) There was no extensive extrapulmonary metastasis (bone metastasis not included in the contraindications after systematic treatment or radiotherapy; for patients with metastasis beyond the lung, inclusion depends on the individual's performance). (3) Patients had no severe coagulation disorders and pulmonary failure. (4) There was a failure of chemotherapy and/or hormone therapy or discontinuation of treatment due to side effects or patient refusal. (5) Each patient underwent biopsies, and 32 of 46 lung lesions were confirmed to be breast metastases by biopsy. (6) A consensus was reached among the members of the multidisciplinary cancer committee, including oncologists, chest surgeons, radiation oncologists, radiologists, and pathologists.

From January 2012 to January 2018, 41 female patients with lung metastases from breast cancer received computed tomography (CT)‐guided percutaneous MWA. Nine patients were excluded for the following reasons: four cases were lost to follow‐up, three cases had extensive extrapulmonary metastasis, and two cases had lung metastases >5 cm in diameter. The remaining 32 patients were included in this study (Table 1).

**TABLE 1:** Characteristics of patients with lung metastasis from breast cancer

Characteristics	*n* (%)
Total number of patients	32
Age (years)	
≤65	23 (71.9%)
>65	9 (28.1%)
Previous chemotherapy	
No	3 (9.4%)
Yes	29 (90.6%)
Previous radiotherapy	
No	24 (75.0%)
Yes	8 (25.0%)
Previous endocrine therapy	
No	18 (56.2%)
Yes	14 (43.8%)
ER	
Positive	15 (46.9%)
Negative	17 (53.1%)
PR	
Positive	15 (46.9%)
Negative	17 (53.1%)
HER2 overexpression	
No	27 (84.4%)
Yes	5 (15.6%)
Extrapulmonary metastasis	
No	27 (84.5%)
Yes	5 (15.6%)
Maximum tumor diameter (cm)	
≤3	25 (78.1%)
3.1−5	7 (21.9%)
Distribution	
Unilateral lung	28 (87.5%)
Bilateral lungs	4 (12.5%)
No. of tumors	
Single	23 (71.9%)
Multiple (two to five)	9 (28.1%)

*Abbreviations*: ER, estrogen receptor; HER2: human epidermal growth factor receptor 2; PR, progesterone receptor.

During MWA, three patients (9.4%) had controllable and isolated bone metastasis, which was not considered a determinant of life expectancy. One case (3.1%) had solitary liver metastasis. Another patient (3.1%) had brain metastases, which had been successfully treated with a gamma knife before MWA. Twenty‐seven cases (84.4%) had no extrapulmonary metastasis.

Thirty‐two women had an average (range) age of 57.3 ± 9.5 (28–76) years. The average (range) maximum diameter was 1.7 ± 0.2 (0.8–4.6) cm. Twenty‐eight cases were single lung metastasis (23 cases were single lesions in unilateral lung and five cases were multiple lesions in unilateral lung) and four cases were double lung metastasis. Thirty‐two breast cancer patients with 46 pulmonary metastases received CT‐guided percutaneous MWA. One patient did not receive any antitumor treatment before ablation, and the others received one or two or three kinds of chemotherapy, radiotherapy, and endocrine therapy. Adjuvant chemotherapy included cyclophosphamide, Adriamycin, capecitabine, and paclitaxel liposomes. Of the 32 patients, 14 received endocrine therapy, including tamoxifen and letrozole. After MWA, all patients were treated by oncologists according to hormone receptor status and follow‐up examination.

### MWA procedure

All patients were treated and followed up under the guidance of multislice CT (Lightspeed 16; GE Healthcare). Before ablation, patients usually were in the lateral, supine, or prone position to scan the lung, and the scanning thickness was about 3–5 mm. After CT scanning, the appropriate scanning layer was selected to determine the puncture angle and depth. The MTC‐3C MWA system (Vison‐China Medical Devices R&D Center, CFDA Certificate No. 20153251978) was used. In general, the chosen ablation power was 40–50 W, and the ablation duration was 3–10 min. For tumors >3.5 cm, two antennas were used for ablation. Immediately after MWA surgery, a CT scan was performed again to assess the tumor size, tumor morphology, and condition of adjacent organs and determine whether there were any complications, such as bleeding and pneumothorax. During operation, blood pressure, heart rate, electrocardiogram, and peripheral blood oxygen saturation were carefully monitored. After MWA, patients entered the ward to observe the changes in vital signs, clinical symptoms, and urine volume.

### Assessment of therapeutic efficacy and follow‐up

The primary technical success was determined in the first follow‐up imaging study after the completion of MWA. To evaluate the effects of ablation, a CT scan was performed for each patient at 1 month after ablation. The primary response rate was the percentage of the target tumor successfully eradicated during initial ablation. The secondary response rate referred to the successful ablation of the tumor after the local progression of the tumor. When the initial follow‐up image showed that the tumor remains at the ablative margin (i.e. the attenuation difference of CT images before and after angiography was ≥20 Hounsfield units), it was called residual unablated tumor. After the initial CT evaluation, a CT scan was performed every 3 months. Local tumor recurrence or progression refers to the appearance of new tumor lesions at the edge of ablation. The local efficacy of MWA was evaluated by one oncologist and two radiologists. The median (range) follow‐up time after MWA was 32 (9–79) months.

### Statistical analysis

Data were analyzed using IBM SPSS statistical software version 25. Data were expressed as the total number (percentage) and average value. The *χ* ^2^ test was used for categorical variables. The Kaplan–Meier method was used to calculate the survival rate and local progression‐free survival rate. For all statistical tests, statistical significance was *p* < 0.05.

## RESULTS

### Clinical outcomes

**FIGURE 1:**
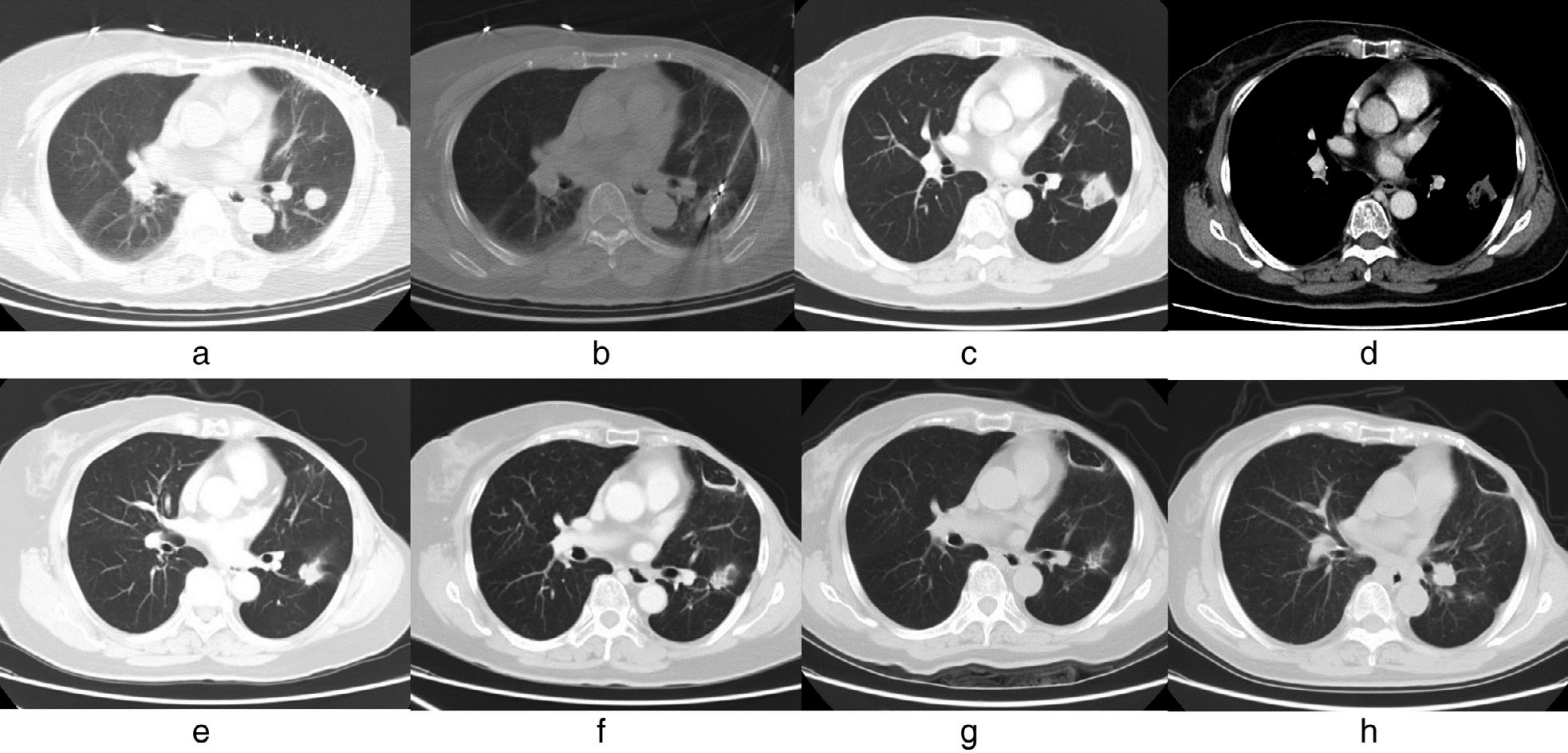
Images of a 74‐year‐old woman who developed left lung metastasis 5 years after breast cancer surgery. (a) The lesion was located in the lower lobe of the left lung with a maximum cross‐sectional diameter of 1.5 × 1.6 cm. (b) The lesion was ablated by microwave. (c and d) One month after ablation, the lesion area was larger than before, but there was no obvious enhancement after the enhanced scan, so complete ablation was considered. (e) At 9 months after ablation, the lesion was smaller than before. (f) At 12 months after MWA, the lesion was significantly smaller. (g) At 24 months after MWA, the lesion continued to shrink. (h) At 40 months after the second ablation, the lesion almost disappeared

All patients were successfully ablated (the technical success rate was 100%). All 32 patients underwent CT scan immediately after operation. Forty‐six lesions showed ground glass‐like exudation and cavities of different sizes in the ablation area. One month after the operation, a CT scan showed that 45 lesions were completely covered by the tumor coagulation area after ablation, and the primary effective rate was 97.8% (45/46) (Figure 1).

During follow‐up, five of 46 lesions had local progression (10.9%), with a median progression time of 10 months. These five lesions belonged to five different patients, and they were all ablated twice. Only one patient's lesion still progressed after the second ablation, and the rest of the patients did not progress during the remaining follow‐up period after repeated MWA. Therefore, the secondary efficacy rate was 80% (4/5). The recurrence rates were 5.1% and 42.8%, respectively. The local recurrence rate of the tumor size >3 cm group was higher than that of the tumor size ≤3 cm group (*p* < 0.001).

### Survival rate and prognostic analysis

**FIGURE 2:**
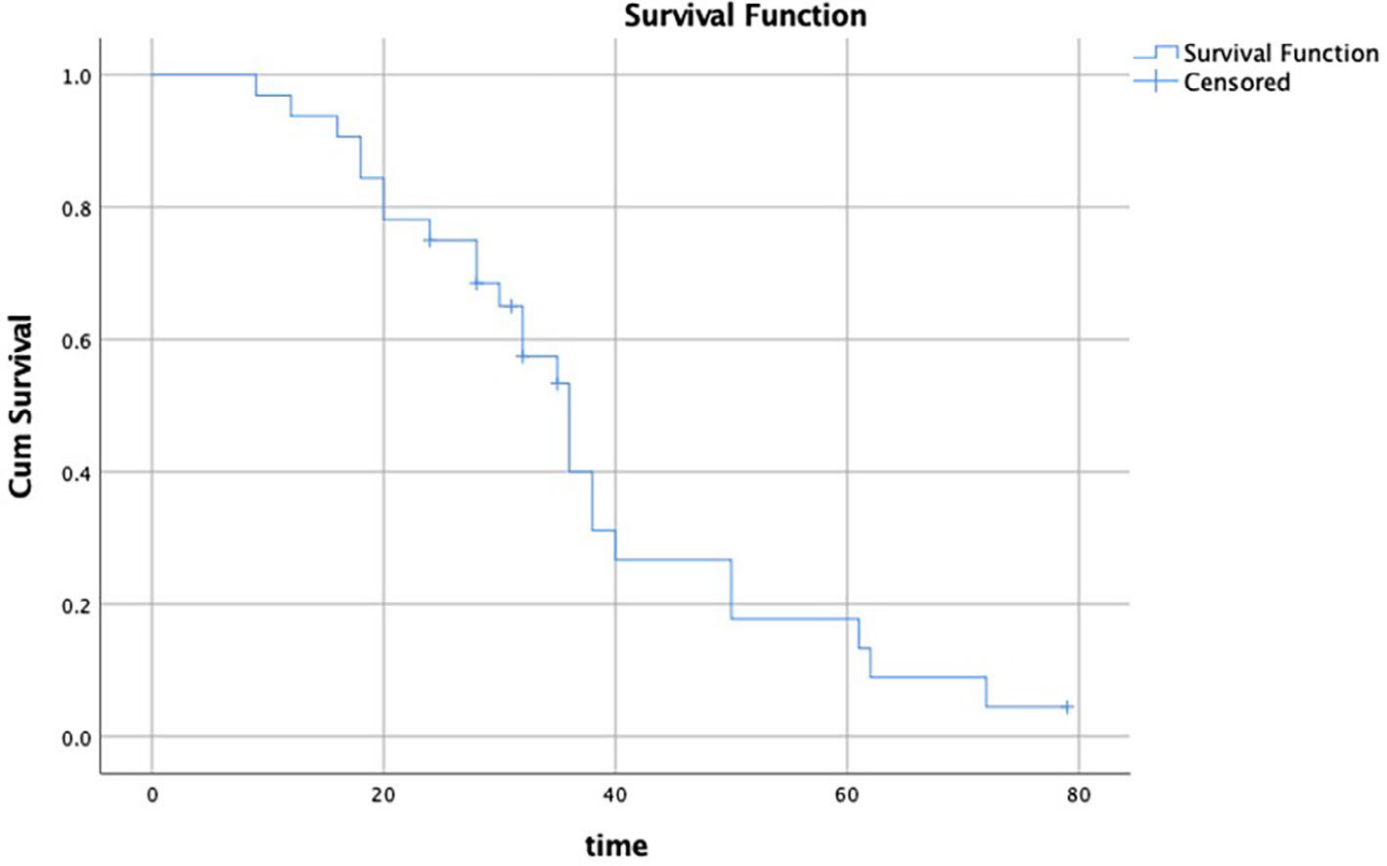
OS rate of 32 patients (46 cases) with lung metastases from breast cancer

The median (range) follow‐up time after MWA was 32 (9–79) months. The 1‐year overall survival (OS) rate was 96.9%, the 2‐year OS rate was 75%, the 3‐year OS rate was 53.3%, and the 5‐year OS rate was 17.8% (Figure 2). The median OS time was 36 months (95% confidence interval [CI] 31.66–40.34).

**FIGURE 3:**
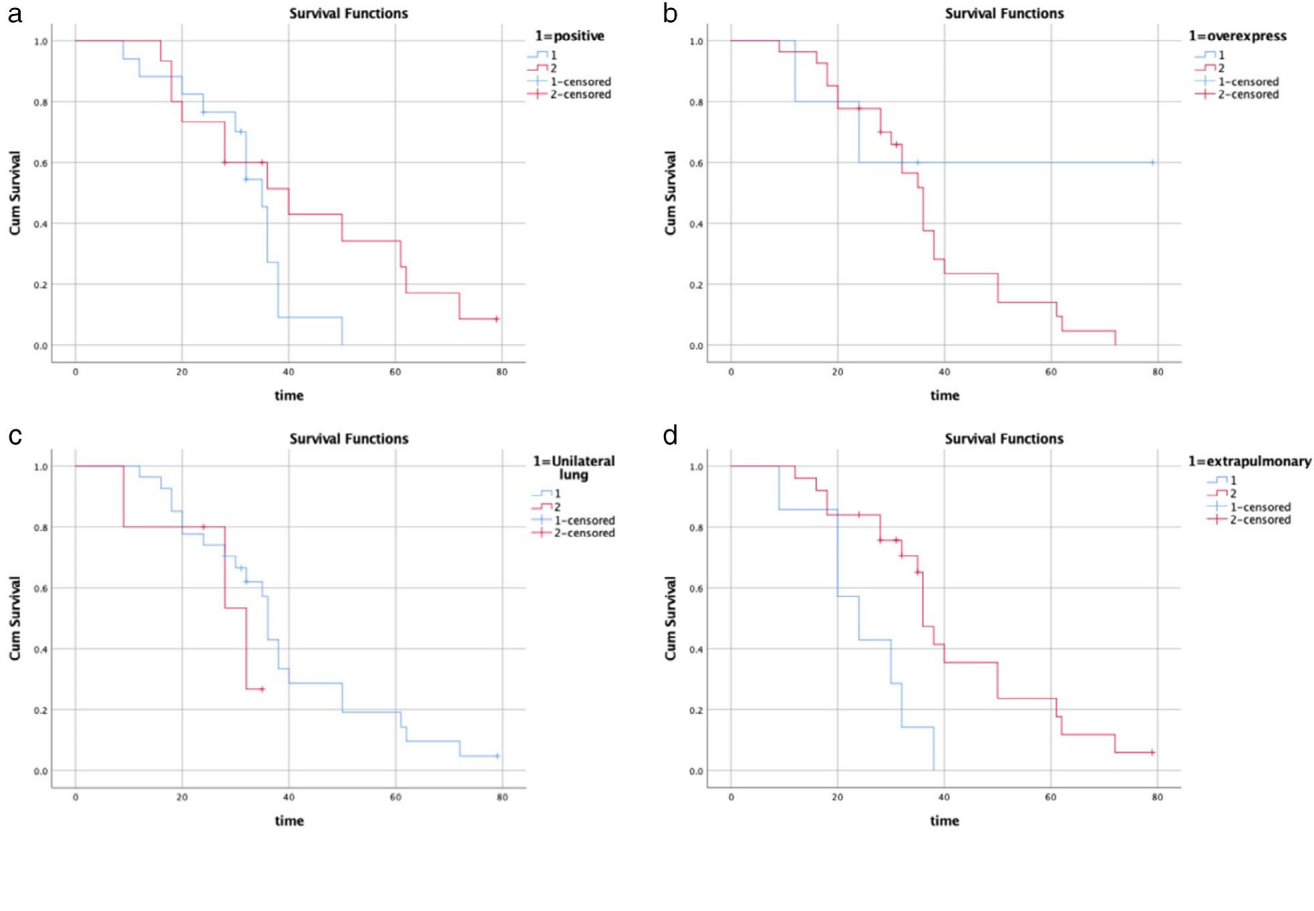
(a) Survival curve of hormone receptor positive (blue line) or negative (red line). There was no significant difference in the median survival time (*p* = 0.137). (b) Survival curve with (blue line) or without (red line) HER2 overexpression: There was no significant difference in median survival (*p* = 0.228). (c) Survival curve of patients with unilateral (blue line) or bilateral (red line) lung metastasis. The difference in survival probability was not statistically significant (*p* = 0.05). (d) Survival curve of patients with (blue line) or without (red line) extrapulmonary metastasis. The estimated median survival time of patients with and without extrapulmonary metastasis was 24 and 36 months, respectively. The difference was statistically significant (*p* = 0.005)

There was no statistically significant difference in survival probability between patients with hormone receptor status positive (*n* = 15) and negative (*n* = 17; log‐rank test, *p* = 0.137; *χ* ^2^ = 2.206; Figure 3a). The median survival times of patients with hormone receptor status positive and negative were 35 months (95% CI 31.24–38.76) and 40 months (95% CI 21.26–58.74), respectively (Table 2).

**TABLE 2:** Outcome of prognostic analysis

Factor	Number of patients	Median survival time (months)	95% CI	*χ* ^2^	*p* value
Hormone receptor status (ER/PR)				2.206	0.137
Positive	15	35	31.24–38.76		
Negative	17	40	21.26–58.74		
HER2 overexpression				1.455	0.228
Yes	5	‐	‐		
No	27	36	31.75–40.25		
Distribution				0.910	0.34
Unilateral lung	28	36	34.59–37.41		
Bilateral lungs	4	32	12.92–51.08		
Extrapulmonary metastasis				7.814	0.005
No	27	36	33.16–38.84		
Yes	5	24	13.74–34.27		

There was no significant difference in survival between patients without human epidermal growth factor receptor 2 (HER2) overexpression (*n* = 27) and patients with HER2 overexpression (*n* = 5; log‐rank test, *p* = 0.228; *χ* ^2^ = 1.455; Figure 3b). The median survival time of patients without HER2 overexpression was 36 months (95% CI 31.75–40.25). For the subgroup with HER2 overexpression, the median survival rate was not calculated because the preconditions were not met (Table 2).

There was no significant difference in survival probability between patients with unilateral lung metastasis (*n* = 28) and patients with bilateral lung metastasis (*n* = 4; log‐rank test, *p* = 0.34; χ^2^ = 0.910; Figure 3c). The median survival times of breast cancer patients with unilateral and bilateral lung metastasis were 36 months (95% CI 34.59–37.41) and 32 months (95% CI 12.92–51.08), respectively (Table 2).

Kaplan–Meier evaluation was performed for breast cancer patients with (*n* = 5) or without (*n* = 27) extrapulmonary metastasis (Figure 3d). The statistical significance of birth survival rate was obtained (log‐rank test, *p* = 0.005; *χ* ^2^ = 7.814). The median survival times of patients with and without extrapulmonary metastasis were 24 months (95% CI 13.74–34.27) and 36 months (95% CI 33.16–38.84), respectively (Table 2).

### Side effects and complications

The common side effects after MWA mainly included pain, postablation syndrome, cough, and pleural reaction (see Table 3). Of the 46 cases of MWA ablation, 12 (26.1%) had intraoperative or postoperative pain, of which nine had mild pain without intervention and three had severe pain requiring active treatment. Postablation syndrome occurred in 10 cases (21.7%). The main symptoms were fever (<38.5°C), fatigue, general discomfort, nausea, and vomiting. There were 13 cases (28.3%) of cough, including nine cases of mild cough and four cases of severe cough. One case (2.2%) had a pleural reaction, mainly manifested as slow heart rate and low blood pressure.

**TABLE 3:** Side effects and complications during and after microwave ablation procedure

Side effects and complications	*n* (%)
Major complications	
Massive pneumothorax (chest tube)	11 (23.9)
Massive pleural effusion (chest tube)	2 (4.3)
Pulmonary infection	2 (4.3)
Minor complications	
Mild pneumothorax (no chest tube)	5 (10.8)
A small amount of pleural effusion (no chest tube)	28 (60.8)
Mild asymptomatic hemothorax	1 (2.2)
Hemoptysis	5 (10.9)
Subcutaneous emphysema	4 (8.7)
Common side effects	
Pain	12 (26.1)
Fever	
<38.5°C	2 (4.3)
≥38.5°C	0 (0)
Fatigue	4 (8.7)
General malaise	2 (4.3)
Nausea	1 (2.2)
Vomiting	1 (2.2)
Cough	13 (28.3)
Pleural reaction	1 (2.2)

The major complications included massive pneumothorax, massive pleural effusion, and pulmonary infection. The minor complications included mild pneumothorax, a small amount of pleural effusion, mild asymptomatic hemothorax, hemoptysis, and subcutaneous emphysema (see Table 2). Among them, 16 cases (34.8%) were pneumothorax and 11 cases needed catheter drainage, including four cases during operation and seven cases 24 h after operation. Pleural effusion occurred in 30 cases (65.2%), and drainage was needed in two cases (4.3%). Hemoptysis was found in five cases (10.9%). Conventional hemostatic drugs can effectively relieve hemoptysis. Mild subcutaneous emphysema occurred in four cases (8.7%). Postoperative pulmonary infection occurred in two cases (4.3%), which were cured after effective antibiotic treatment. Mild asymptomatic hemothorax was found in one case (2.2%), which was naturally absorbed without special treatment. No ablation‐related death occurred during operation and within 30 days after the operation.

## DISCUSSION

In this study, 45 of 46 lesions (97.8%) were completely ablated. For tumors ≤3 cm, the complete ablation rate was 100%, indicating that MWA effectively treated lung metastases from breast cancer. Local progression after ablation has always been one of the difficulties in clinical treatment. In this study, five of 46 lesions (10.9%) developed local progression. There were two recurrent lesions with a maximum diameter of ≤3 cm and three cases of recurrent lesions >3 cm. The local recurrence rate of a tumor with a diameter of ≤3 cm was significantly lower than that of a tumor with a diameter of >3 cm (*p* < 0.001). It could be seen that the size of the lesion was an important factor of local progression.

Some studies have shown that although metastatic breast cancer was a systemic disease, local treatment could improve the survival time. MWA was performed in patients with lung metastases from breast cancer.^13^, ^14^ It has been reported^3^ that the median survival time of breast cancer patients with lung metastasis was only 22 months, but data from this study showed that the median survival time could reach 37 months, suggesting that MWA may effectively improve the survival rate of patients with lung metastases from breast cancer. Studies have shown that surgical resection of single lung metastasis in breast cancer patients could improve the survival rate, and the 5‐year survival rate was 54.5%.^15^ Some studies have also pointed out that the median survival time after resection of lung metastases is 32–97 months, and the 5‐year survival rate is 27–80%.^6^ According to the International Lung Metastasis Registry, the median OS and 5‐year OS rates in patients undergoing surgical resection of lung metastases from breast cancer were 37 months and 38% in the R0 group and 25 months and 18% in the incomplete resection group, respectively.^16^ There was no significant correlation between solitary metastasis and survival rate. In this study, the 1‐, 3‐, and 5‐year OS rates were 96.9%, 53.3%, and 17.8%, respectively. Compared to the data of surgical resection, the 5‐year survival rate in this study was only 17.8%. It may be related to the number of lung metastases, disease progression, and distant metastasis of other organs. It is suggested that the overall prognosis of breast cancer patients with lung metastasis is poor, especially in patients with extrapulmonary metastasis.

At present, more and more clinical studies have confirmed the safety of MWA in the treatment of pulmonary malignancies.^17^ In this study, no patient died during and 30 days after ablation. The common side effects of MWA were pain (26.1%), cough (28.3%), and postablation syndrome (21.7%). The major complications included massive pneumothorax (23.9%), massive pleural effusion (4.3%), and pulmonary infection (4.3%). Patients with pneumothorax and pleural effusion were improved after positive drainage. Pulmonary infection can be effectively cured by appropriate antibiotics. There were no reported life‐threatening cases.

There are still some limitations in this research. As a retrospective study, data were only based on small, heterogeneous single‐institution series reports and there was a lack of prospective, randomized trials to prove the advantages of this treatment compared to conventional treatment. Future studies should adopt a larger sample size and control these changes. A prospective, randomized, controlled study design can be used to compare MWA to surgical resection, radiotherapy, or chemotherapy.

## CONCLUSIONS

MWA is safe and effective in the treatment of lung metastases from breast cancer. However, there are still many problems, such as whether MWA can replace surgery and how to combine MWA with other treatment methods to improve the treatment effects.

## CONFLICT OF INTEREST

The authors declare that they have no conflict of interest for this article.
